# A “Wearable” Test for Maximum Aerobic Power: Real-Time Analysis of a 60-m Sprint Performance and Heart Rate Off-Kinetics

**DOI:** 10.3389/fphys.2017.00868

**Published:** 2017-11-01

**Authors:** Jorge L. Storniolo, Gaspare Pavei, Alberto E. Minetti

**Affiliations:** Laboratory of Locomotion Physiomechanics, Department of Pathophysiology and Transplantation, University of Milan, Milan, Italy

**Keywords:** maximum aerobic power, heart rate, off kinetics, sprint running, smart devices

## Abstract

Maximum aerobic power (${\dot{V}O}_{2peak}$) as an indicator of body fitness is today a very well-known concept not just for athletes but also for the layman. Unfortunately, the accurate measurement of that variable has remained a complex and exhaustive laboratory procedure, which makes it inaccessible to many active people. In this paper we propose a quick estimate of it, mainly based on the heart rate off-kinetics immediately after an all-out 60-m sprint run. The design of this test took into account the recent availability of wrist wearable, heart band free, multi-sensor smart devices, which could also inertially detect the different phases of the sprint and check the distance run. 25 subjects undertook the 60-m test outdoor and a ${\dot{V}O}_{2peak}$ test on the laboratory treadmill. Running average speed, HR excursion during the sprint and the time constant (τ) of HR exponential decay in the off-kinetics were fed into a multiple regression, with measured ${\dot{V}O}_{2peak}$ as the dependent variable. Statistics revealed that within the investigated range (25–55 ml O_2_/(kg min)), despite a tendency to overestimate low values and underestimate high values, the three predictors confidently estimate individual ${\dot{V}O}_{2peak}$ (*R*^2^ = 0.65, *p* < 0.001). The same analysis has been performed on a 5-s averaged time course of the same measured HR off-kinetics, as these are the most time resolved data for HR provided by many modern smart watches. Results indicate that despite of the substantial reduction in sample size, predicted ${\dot{V}O}_{2peak}$ still explain 59% of the variability of the measured ${\dot{V}O}_{2peak}$.

## Introduction

In the last few decades we assisted to a growing interest toward personally keeping one's health in a better shape, a condition that would enrich the entire life and likely prevents early deterioration of many body functions. This passes, among others, through the development and maintenance of a maximum oxygen consumption (${\dot{V}O}_{2peak}$) higher than for a sedentary. However, portable professional metabographs are out of reach for most of the athletes, not to mention the laymen, who represents the vast majority of the potential audience in the need to periodically check the aerobic fitness level.

At the same time, the progress in terms of portable technology (tablets, “smart” phones, bracelets/bands and watches) makes us move equipped with a redundancy of sensors. In addition to the ubiquitary camera, most of the devices bring GPSs, accelerometers, gyroscopes, magnetometers, proximity sensors and, most recently, infrared emitter/detector LED systems to measure heart rate (HR) in real-time. Although not all of them provide data accurately and precisely enough to compete with the analogous laboratory equipment (Chowdhury et al., 2017), their improvement is just a matter of time and scenarios for new and different biomedical tests could be certainly hypothesized to be implemented in the near future.

Submaximal metabolic effort such as walking, running, hiking, swimming at moderate speed has been classically included in the activity monitor function of “smart” portable/wearable devices. The estimate of burned calories is obtained from short term average HR, average speed and from accelerometry-based recognition of locomotion type (Chowdhury et al., 2017).

Differently, no estimate of ${\dot{V}O}_{2peak}$ from smart devices has been implemented so far, to the authors' knowledge. Potential reasons for this is, as mentioned, the infancy of wearable sensor technology that strives to compete with professional analogs. Our challenge in the present investigation was to design a simple test exploiting sensors already incorporated in smart watches/bracelets.

The idea originated from transport engineering: race car engines increase and decrease rpm (revolutions per minute, a “sound” particularly apparent when gear is disengaged) much faster than in a normal car. In the biological realm we face a similar phenomenon: athletes display a faster ${\dot{V}O}_{2}$ increase (at the start of a heavy exercise) and decrease (during the recovery) than sedentary subjects (for a review see Jones and Poole, 2005; Rossiter, 2011). HR is a fundamental determinant of ${\dot{V}O}_{2}$ on- and off-kinetics, since its time course closely mimics the changes in gas exchange (Hickson et al., 1978; Hagberg et al., 1980; Norris and Petersen, 1998). Thus, similarly to engine rpm, HR kinetics is expected to be faster the higher the metabolic power of human engine (Darr et al., 1988; Sugawara et al., 2001; Otsuki et al., 2007; Ostojic et al., 2010, 2011; Watson et al., 2017). This applies to other important kinetics, such as the enzymatic chain (Timmons et al., 1998), within the whole metabolic/mechanical “turn on/off” process of muscular exercise.

A very short maximal sprint (60-m) was adopted in order to design a quick test that could be performed in a non-specialized environment: only rubber soles and a short straight path, in addition to the smart watch, would be necessary. We decided to use only the HR off-kinetics because even more professional HR sensors (i.e., the thoracic belt) have troubles to detect just the heart signal when many other muscles in the body are intensively activated, as during maximal propulsion.

Aim of the study was to propose a simple methodology and algorithm predicting individual aerobic fitness, and check its adherence to experimentally measured ${\dot{V}O}_{2peak}$ values. This test could be implemented in many smart wearable devices and would certainly benefit from the inevitable improvement in sensor technology.

## Materials and methods

### Subjects

Twenty-five subjects (7 women and 18 men, 25.0 ± 5.0 year, 1.77 ± 0.08 m height, 71.4 ± 8.6 kg body mass; mean ± SD) took part in the study; they were physically active subjects involved either in recreational activity or in amateur sport activity with a maximum of four sessions per week. The study was approved by the Ethics Committee of the University of Milan (031511), and participants, after becoming aware of the potential risks involved in the experimental sessions, gave written informed consent.

### Experimental protocol

Subjects performed two different tests in different days separated by a minimum of 48 h: a 60-m maximal sprint accomplished on an outdoor athletic track and an incremental exercise test for the determination of ${\dot{V}O}_{2peak}$ in the laboratory. Participants were instructed to arrive at the experimental session in a rested and fully hydrated state and to avoid strenuous exercise in the 24 h preceding each testing session. In addition, they were told to avoid alcohol (24 h) and caffeine (6 h) intake before the exercise test.

The first session consisted of a 60-m maximal sprint trial preceded by a short warm-up (5 min with jogging and stretching) and 10 min of resting period (5 min in a seated and 5 min in a standing position). Heart rate (HR) was recorded beat-by-beat throughout all phases of the sprint test (rest, running and 5 min of recovery) by a heart rate monitor with transmitter belt (Polar S410, Kempele, Finland). All tests were performed at the same time of day (10–11 a.m.) to limit the influences of circadian rhythm on muscle performance and heart rate response/variability. 60-m sprint duration was recorded by using a manual stopwatch and the average running speed (*v*_*test*_, m^.^s^−1^) was obtained. Subjects were encouraged to accomplish their best performance.

Peak aerobic power ($\dot{V}O_{2peak}$) was determined with an incremental running test performed on a treadmill (Ergo LG, Woodway). After 10 min of standing resting period, the protocol began with subjects running at 9 km^.^h^−1^ for 4 min, then the belt speed was increased by 1 km^.^h^−1^ every minute until volitional exhaustion. Pulmonary ventilation ($\dot{V}E$, BTPS), O_2_ consumption ($\dot{V}O_{2}$), and CO_2_ output ($\dot{V}$CO_2_), both STPD, were determined breath by breath by a portable metabograph (K4b^2^, Cosmed, Italy). $\dot{V}O_{2peak}$ values were taken as the highest 30 s average $\dot{V}O_{2}$ value attained before the subject's volitional exhaustion. Respiratory exchange ratio (RER) was calculated as the ratio of $\dot{V}$CO_2_ to $\dot{V}O_{2}$. At rest and at various times (5, 7, and 9 min) during recovery, 0.6 μL of capillary blood was obtained from a preheated earlobe for the determination of blood lactate concentration ([La]_b_) (Lactate Plus, Nova Biomedical).

### Heart rate kinetics

**Figure 1:**
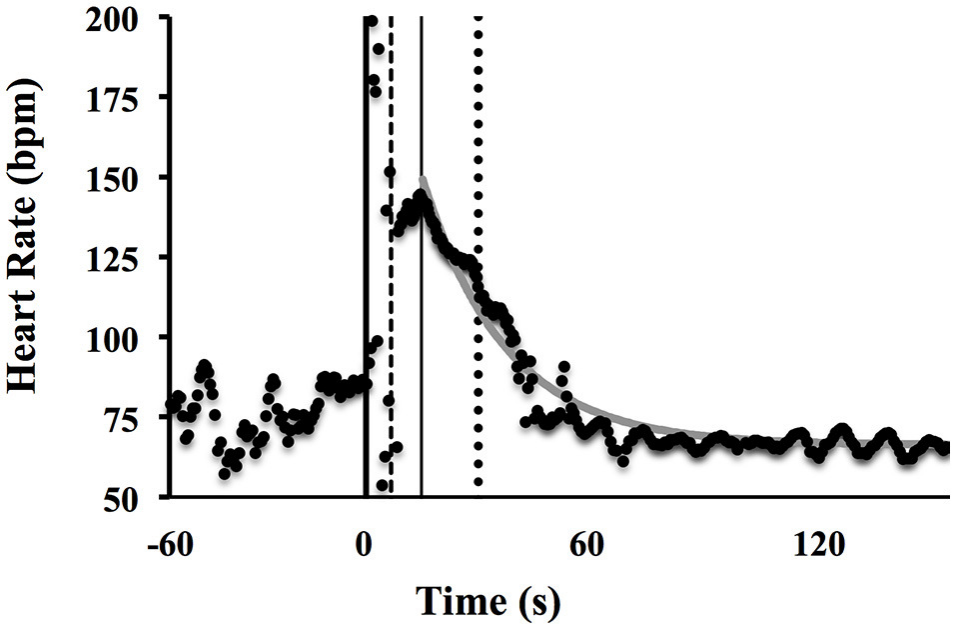
Example of a representative heart rate time course from rest to recovery of the maximal sprint test. The thick vertical line coincides with the start of the sprint, the dashed vertical line with the end of the sprint, the thin vertical line represents the start decay of HR, while the dotted line expresses the τ_*off*_ and the gray continuous curve represents the mono-exponential best fit.

Heart rate off-kinetics (HR decrease after 60-m sprint) was modeled according to a mono-exponential function of time by using a Least Squares Method (minimizing the sum of squared vertical distances between experimental points and the exponential curve):

(1) $$\begin{array}{r} HR\left(t\right)=HR_{baseline}+Ampl\cdot e^{\left(-t/\tau_{off}\right)} \end{array}$$

where *HR*_*baseline*_ is the average of HR (bpm) during the last 60 s of the recovery period; *Ampl* is the asymptotic amplitude for the exponential term (maximal HR values − HR_baseline_, bpm); τ_*off*_ is the time constant (s) of the exponential, i.e., the time from the end of the sprint to reach 27% of HR maximum excursion [which corresponds to *HR* = *HR*_*baseline*_ + *Ampl* (1–63%)]. The velocity of HR decays after the sprint (*v*_*off*_, s^−1^) was inferred as the reciprocal of τ_*off*_. Also, the heart rate range from the sprint start to the beginning of the off-kinetics phase (ΔHR) was calculated (Figure 1). All data have been analyzed with purposely written LabView programs (release 13, National Instruments).

### Statistics

Data are presented as mean ± standard deviation (SD). Multiple linear regression analysis was adopted to explain the variance of the individual $\dot{V}O_{2peak}$, based on independent variables *v*_*test*_, *v*_*off*_ and ΔHR. Linear regressions were used to analyse correlations between variables and residuals of predicted ($\hat{\dot{V}}O_{2peak}$) vs. measured $\dot{V}O_{2peak}$. The Standard Error of the Estimate (SEE) was calculated to measure the accuracy of the prediction and to compare it to other published predictors of $\dot{V}O_{2peak}$. Statistical significance was granted at *p* ≤ 0.05. Statistical analysis was performed by using SPSS v20 (IBM, USA).

## Results

$\dot{V}O_{2peak}$ range was 29.1–56.6 ml^.^kg^−1.^min^−1^ (mean ± SD, 42.5 ± 8.7 ml^.^kg^−1.^min^−1^). Peak net blood lactate concentration after the incremental test was 8.5 ± 1.3 mM. All groups attained maximal HR values corresponding to 95% of the age predicted maximum and RER values > 1.1. Thus, taking into account also [La]_b_ peak values, it can be assumed that subjects reached the maximum exercise capacity.

**Figure 2:**
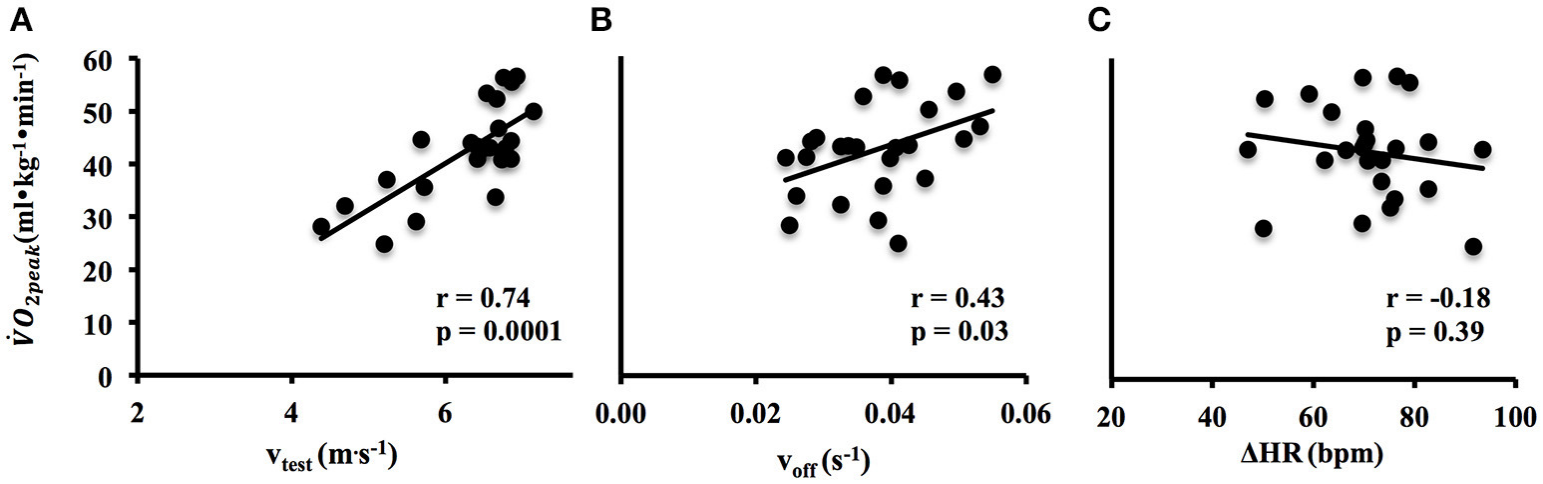
Relation between $\dot{V}O_{2peak}$ and *v*_*test*_ **(A)**, *v*_*off*_ **(B)**, and ΔHR. **(C)** are illustrated.

The *v*_*test*_ and *v*_*off*_ were significantly related to $\dot{V}O_{2peak}$ (*r* = 0.74, *p* < 0.001; *r* = 0.43, *p* = 0.03, respectively) (Figures 2A,B), whereas ΔHR was not related to $\dot{V}O_{2peak}$ (*r* = −0.18, *p* = 0.39) (Figure 2C).

**Figure 3:**
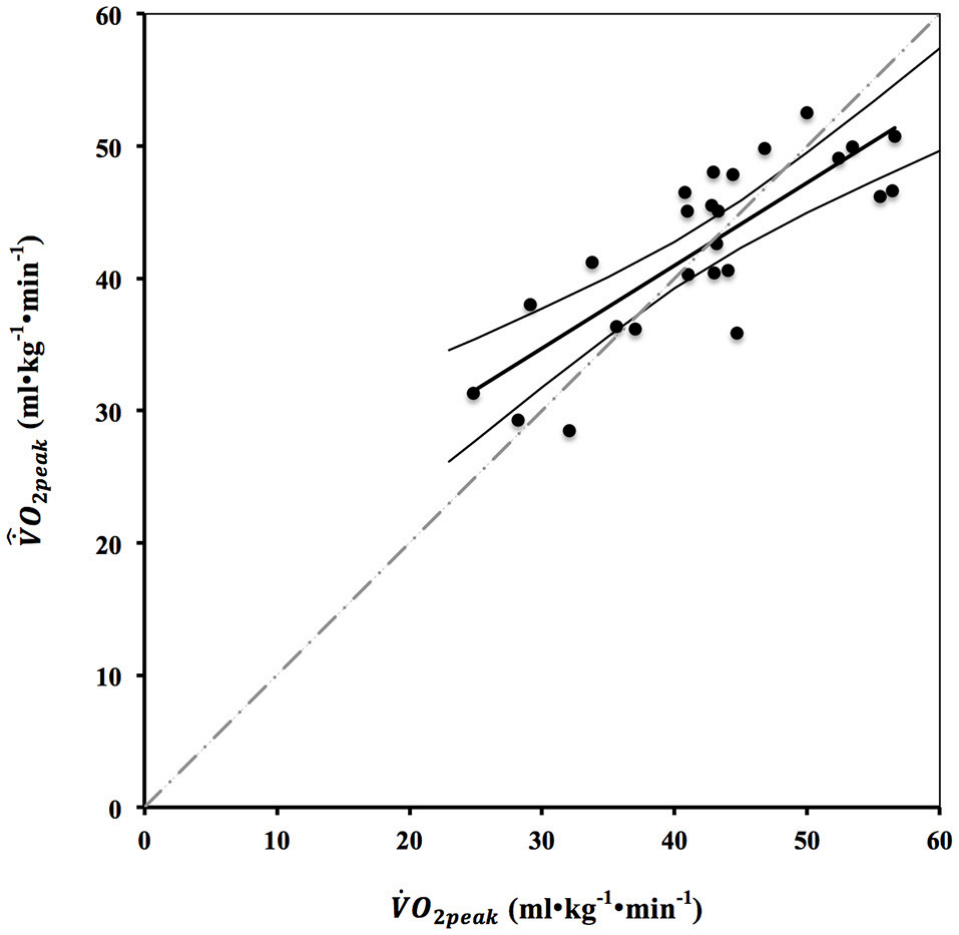
Relation between measured $\dot{V}O_{2peak}$ and predicted $\hat{\dot{V}}O_{2peak}$ estimated from multiple linear regression $\hat{\dot{V}}O_{2peak}$ = 7.46·*v*_*test*_ + 261.4·*v*_*off*_ − 0.19·ΔHR. Trend line (thick black line) expresses the linear regression between the variables ($\hat{\dot{V}}O_{2peak}$ = 0.62 $\dot{V}O_{2peak}$ + 15.9; *r* = 0.80). The thin black lines are the confidence interval (95%) of the trend line, while the dashed gray line is the identity line.

Multiple regression analysis ($\dot{V}O_{2peak}=\;7.46\cdot v_{test}+\;261.4\cdot v_{off}\;-\;0.19\;\cdot \;\Delta$HR) showed that a linear combination of *v*_*test*_, *v*_*off*_ and *HR* from the sprint test explained 65% of the $\dot{V}O_{2peak}$ variance (*R*^2^ = 0.65, *p* < 0.001) (Figure 3).

**Figure 4:**
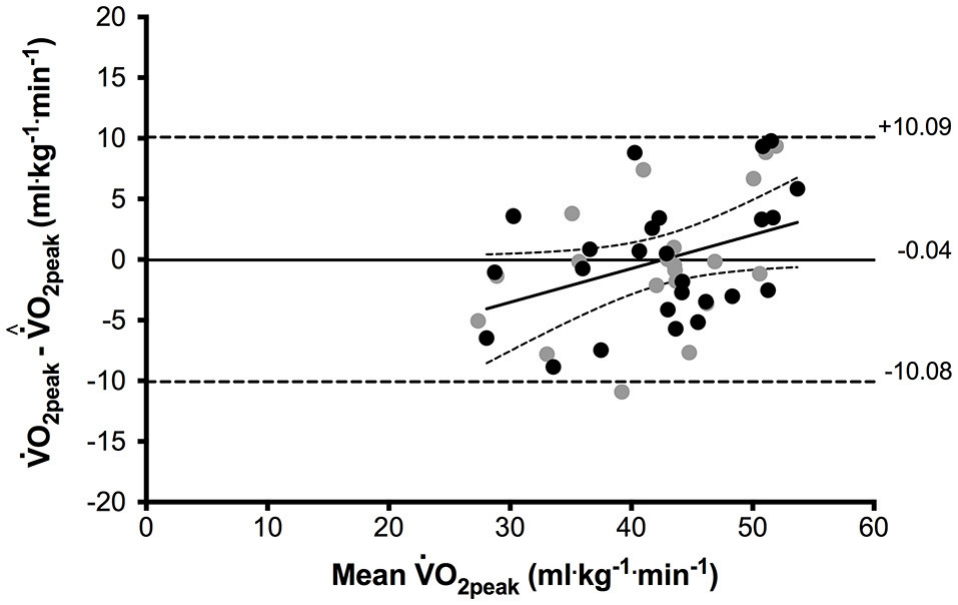
Bland-Altman plot of $\dot{V}O_{2peak}$ difference (measured − predicted values) vs. mean $\dot{V}O_{2peak}$ (black circles). Solid line (average bias = −0.04 ml^.^kg^−1.^min^−1^); dashed line indicates 95% limits of agreement. The trend line equation expresses y = 0.28x − 11.9, with *r* = 0.40 (*p* = 0.04) with confidence intervals (95%). Gray circles: predicted values based on 5 s average of HR time course (see section Discussion).

A paired *t*-test did not show significant difference (*p* = 0.97) between measured $\dot{V}O_{2peak}$ and the predicted value ($\hat{\dot{V}}O_{2peak}$) and the SEE was 5.28 ml^.^kg^−1.^min^−1^. Figure 4 shows the Bland-Altman plot $\dot{V}O_{2diff}\left(=\hat{\dot{V}}O_{2peak}-\dot{V}O_{2peak}\right)$ vs. mean $\dot{V}O_{2peak}$, both for a beat-to-beat analysis and for a 5 s average of HR off-kinetics (see below).

## Discussion

The idea behind this investigation has been to find a simple and short test that could reasonably predict individual $\dot{V}O_{2peak}$, based on signals from sensors that constitute the current “equipment” inside mobile/smart devices (phones or watches).

**Figure 5:**
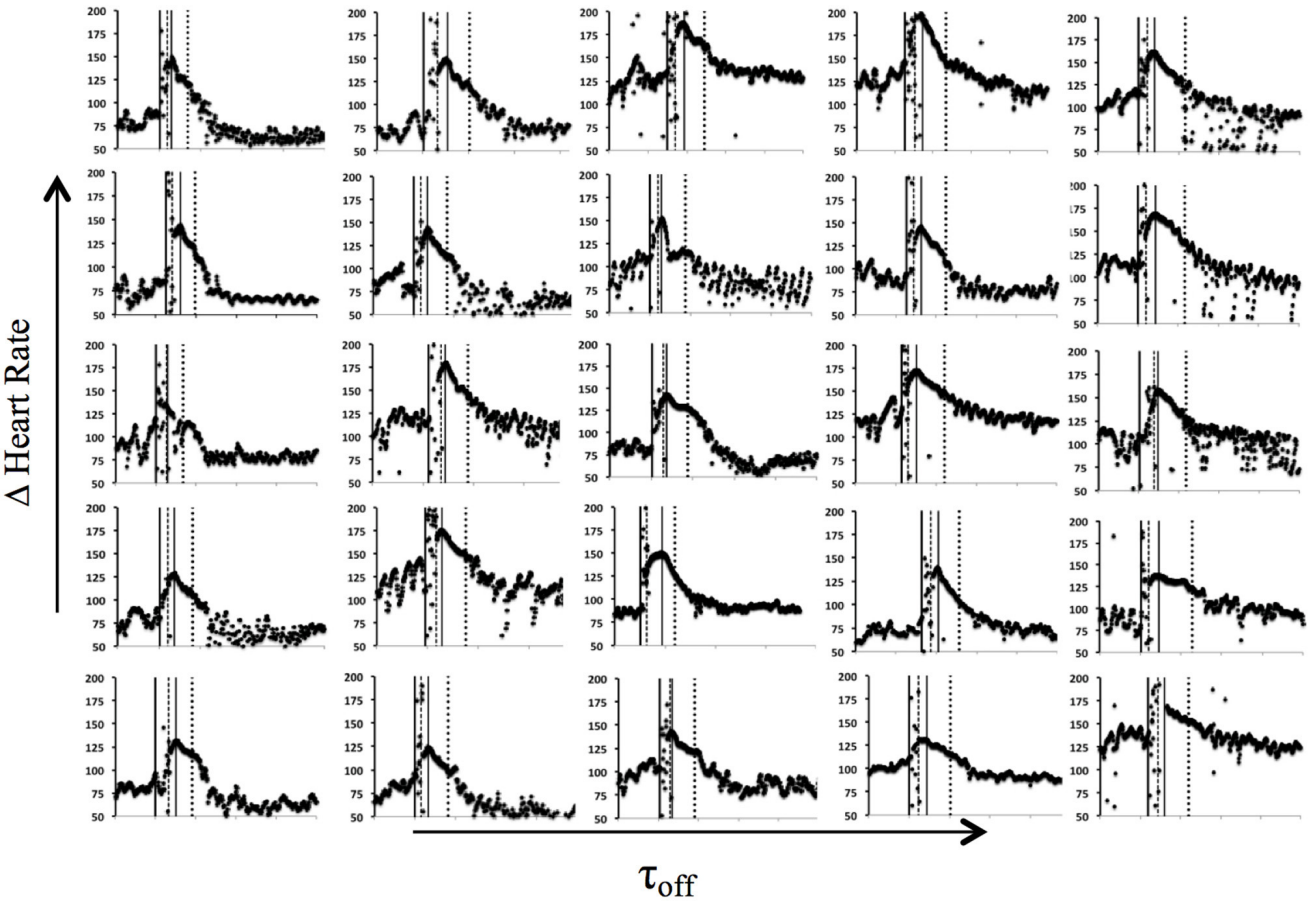
Heart rate recording of each participant (*n* = 25) during the sprint test and their respective markers: thick black vertical line as start of the sprint, dashed vertical line representing end of the sprint, thin vertical black line denotes the start decay of HR, and dotted line expresses the τ_*off*_.

The software algorithm has been designed as to use the most meaningful part of the post-sprint HR time course: it was noted that signal is often still increasing or almost constant before starting the decay toward the resting value (Figures 1, 5). Thus, in order to better quantify HR off-kinetics, a routine trimmed the data and fed the statistical procedure (exponential regression Least Squares Method) with just-decay values. We did not analyse the on-kinetics because, as expected, during the sprint, HR values are scattered (see the data between the first two vertical lines in Figures 1, 5) presumably due to both the interferences of the contracting thoracic muscles and of the belt vibrations on the ECG signal. However, as an indicator of the on-kinetics the overall HR variation from rest baseline to the beginning of the off-kinetics (ΔHR) was included in the model.

The Multiple Regression has been designed to correlate a measure of metabolic power (= metabolic work/time, $\dot{V}O_{2peak}$) to three predictors: as two of them originally have units with time at the denominator (*v*_*test*_ and ΔHR), we decided to transform τ_*off*_ into $v_{off}=\frac{1}{\tau_{off}}$, to increase the “linearity” of their statistical effect.

**Figure 6:**
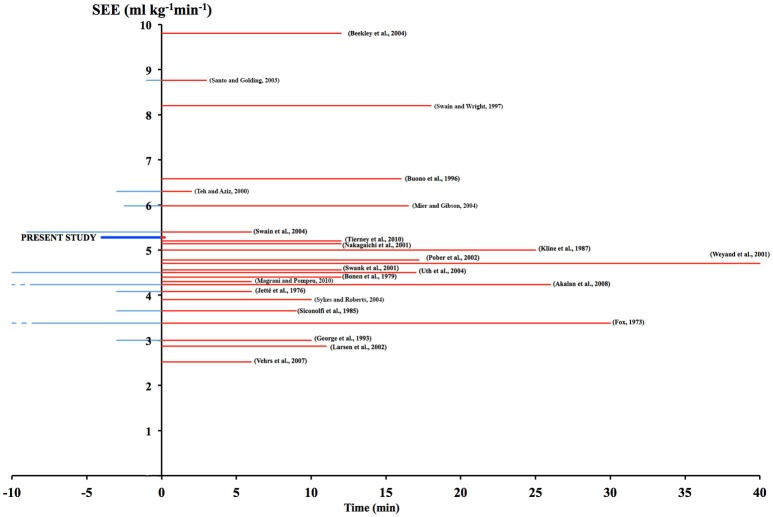
Accuracy of the $\dot{V}O_{2peak}$ prediction, as SEE (ml^.^kg^−1.^min^−1^), is presented in relation to average protocol duration (*t*, min) clustered in exercise (red positive bars) and rest (blue negative bars) time. Present data is shown as thick lines. SEE of other predictive equations on submaximal protocols are shown for comparison with their bibliographic reference. A more detailed discussion about the quoted investigations can be found in Sartor et al. (2013) review.

Despite of the short duration and the simplicity of the test, its reliability in predicting the “real” $\dot{V}O_{2peak}$ values has been witnessed by the significant correlation of Multiple Regression and by an acceptable standard error of the estimate (also in relation to previous literature, see Figure 6 and below). Among the predictors, when individually compared to $\dot{V}O_{2peak}$, only ΔHR does not significantly correlate (albeit negatively) with $\dot{V}O_{2peak}$ (see Figure 2C). This can be ascribed to two contrasting effects: (1) ΔHR should be higher in highly fit subjects due to their faster on-kinetics, and (2) highly fit subjects (as shown by a significant correlation in Figure 2A) run the same 60-m in a shorter time, thus not allowing HR to reach a high value. However, the regression made just by *v*_*test*_ and *v*_*off*_ as variates explains a smaller portion of the experimental $\dot{V}O_{2peak}$ variance (55%), witnessing the value of ΔHR in the multiple regression model.

The HR off-kinetics (*v*_*off*_ Figure 2B) is positively related to $\dot{V}O_{2peak}$: fitter subjects showed a faster decay; this result is in line with previous literature (Darr et al., 1988; Yamamoto et al., 1991; Sugawara et al., 2001; Carnethon et al., 2005; Giallauria et al., 2005; Ostojic et al., 2011; de Mendoca et al., 2017). HR off-kinetics is characterized by a coordinated interaction of parasympathetic re-activation and sympathetic withdrawal (Perini et al., 1989; Imai et al., 1994; Dewland et al., 2007; Borresen and Lambert, 2008; Maeder et al., 2009; Daanen et al., 2012) and it seems that in trained subjects the adaptations in the efferent parasympathetic pathway could accelerate the vagus-mediated heart rate recovery (Imai et al., 1994; Dewland et al., 2007). On the other hand Hagberg et al. (1979) found that this faster recovery was not related to a more rapid recovery of the sympathetic response to exercise. Exercise intensity and type have been shown to influence the speed/tau of HR off-kinetics due to different energetic contribution and released metabolites during exercise and recovery (Pierpont et al., 2000; Buchheit et al., 2007; Borresen and Lambert, 2008; Al Haddad et al., 2009; Nakamura et al., 2009; do Nascimento Salvador et al., 2016). Such heterogeneity in exercise related factors and different indexes used for defining the off-kinetics make a comparison of decays among different studies quite troublesome.

In the present study, subjects performed a shorter effort than those present in literature, reached a maximal HR of 150 ± 20 bpm, which is the 79% of the maximal HR of the incremental test, in about 20 s. At the beginning of the exercise, the onset of HR is characterized by the fast vagal withdraw and the slower sympathetic activation. Even if our exercise duration was very short it seems that both mechanisms would have been activated in order to reach (and then recover from) the 79% of the maximal HR.

As shown in Figures 3, 4, the Multiple Regression overestimates and underestimates measured values at low and high $\dot{V}O_{2peak}$, respectively. The predictive equation was verified with a random sampling approach. From the whole sample (*n* = 25), 12 subjects were randomly extracted and used as a new control group for the predictions of the multiple regression that was performed on the other 13 subjects. This process was performed 35 times (taking care of avoiding duplicated group composition). We obtained 35 new predictive equations and average discrepancies between the measured and predicted $\dot{V}O_{2peak}$. The mean SEE was 6.31 ± 0.82, similar to 5.28 obtained from processing the whole sample.

Although we used lab-quality sensor technology, the implementation of the proposed test on consumer wearables could manage the whole experimental protocol locally: continuous beat-by-beat HR would be used both to monitor/warn the subjects on the most appropriate time at which to start the 60-m sprint (i.e., when a rest steady state is reached) and to collect the recovery phase. GPS and 3-axis accelerometers could provide where and when, respectively, the 60-m sprint started and ended, from which the overall distance traveled can be checked and the average speed calculated.

The use of a “traditional” thoracic belt sensor (Polar S410, Kempele, Finland) has been driven by the need of the most accurate, beat-by-beat HR sensor. There is no such a capability, so far, in most consumer wrist-wearable devices. Even Apple Watch (Apple Inc., California, USA), which has been mentioned for a very high reliability in processing physiological data during physical exercise (Chowdhury et al., 2017; Wang et al., 2017), does not output beat-to-beat intervals. Blood oxygenation pulsations (photo-plethysmography) are detected by photodetectors measuring the bounced back infrared light emitted by LED diodes located between wrist and watch. The fluctuations in blood color absorption due to the local volume changes are measured resulting in HR data. A few studies have emphasized the accuracy of these wrist-worn devices (Spierer et al., 2015; Wallen et al., 2016; Chowdhury et al., 2017; Wang et al., 2017) during rest and exercise. Nevertheless, no system at present seems to be confident enough to deliver single beat interval/frequency, probably due to motion induced artifacts, a problem that could be solved by sensor redundancy and/or signal processing enhancing signal-to-noise ratio.

Current technology confines the time resolution of most of those devices (Parak et al., 2015) to about 5 s, within which an average heart frequency is computed. Although our investigation is particularly meant for next, beat-by-beat sensors, we tested the predictive ability of the proposed algorithm when HR data was provided at 0.2 Hz (as in the actual versions). This was achieved by manipulating the recorded single-beat sequences as to obtain an average value every 5 s. In Figure 4 gray points reflect the approximation involved in using 5 s average HR data, which resulted quite similar to single-beat regressions (*R*^2^ = 59%).

The proposed, indirect $\dot{V}O_{2peak}$ test is certainly not meant to replace the usual direct metabolic measurements and protocols done in a research or clinical laboratory setting, where a much higher accuracy is required in the assessment of subject/athlete's aerobic fitness. By using a portable smart device with multiple sensors and the suggested algorithm, a handy $\dot{V}O_{2peak}$ estimation is at reach for individuals who could later decide whether or not to deepen the awareness of their health status. However, when compared with many other $\dot{V}O_{2peak}$ predicting submaximal protocols done on similar subjects, based on different physical activities and with much longer exercise duration (thus more distressful conditions, quoted in Figure 6), the SEE was quite similar: 5.28 for the 60-m sprint vs. 4.63 ± 1.58 (average) of the literature.

The proposed test leaves space for improvement: (a) HR kinetics are known (Astrand et al., 1986) to be affected by a number of conditions (age, body and ambient temperature, over-training, altitude, fatigue, hydration, etc.) here not taken into account, (b) off-kinetics only have been considered, but new processing techniques (e.g., Salehizadeh et al., 2016) could allow to include HR on-kinetics to better infer $\dot{V}O_{2peak}$, and (c) new refined modeling approaches (e.g., Zakynthinaki, 2015) could help to incorporate in the algorithm and detect slightly differences in the delayed off-kinetics start that could better estimate the fitness level, (d) a greater sample size with inter-subject repeatability could enhance the power of the predictive equation.

Results from the current investigation encourage to develop new simple methods to infer individual physiologic variables by exploiting the current and next portable technology. Watches and bracelets are the perfect candidates, with respect to smart phones, because of their small size and the increased computational power capable to sample and process the data on-board, with no immediate need of external connection.

## Conclusion

A simple and short test (60-m sprint run) could reasonably predict individual $\dot{V}O_{2peak}$ based on the heart rate off-kinetics immediately after the sprint. This test can be easily managed by all individuals with the new wrist wearable, heart band free, multi-sensor smart devices and the proposed algorithm.

## Author contributions

AM conceived the study, JS and GP collected data, JS, GP, and AM analyzed data, wrote the manuscript and revised the final version.

### Conflict of interest statement

The authors declare that the research was conducted in the absence of any commercial or financial relationships that could be construed as a potential conflict of interest.
